# Supramolecular Assembly of Cell Wall Anisotropic Scatterers in Triticale Root Apex Reflects Aluminum Stress Response in Contrasting Genotypes

**DOI:** 10.3390/ijms262311519

**Published:** 2025-11-27

**Authors:** Małgorzata R. Cyran, Krystyna Rybka, Agnieszka Niedziela, Marek J. Potrzebowski, Sławomir Kaźmierski

**Affiliations:** 1 Plant Breeding and Acclimatization Institute—National Research Institute, Radzikow, 05-870 Blonie, Poland; k.rybka@ihar.edu.pl (K.R.); a.niedziela@ihar.edu.pl (A.N.); 2 Centre of Molecular and Macromolecular Studies Polish Academy of Sciences, Sienkiewicza 112, 90-363 Lodz, Poland; marek.potrzebowski@cbmm.lodz.pl (M.J.P.); slawomir.kazmierski@cbmm.lodz.pl (S.K.)

**Keywords:** triticale (× *Triticosecale* Wittmack), root cell wall polysaccharides, aluminum tolerance, multi-detection HPSEC, anisotropic light scatterers

## Abstract

Acid soil aluminum (Al) considerably reduces crop productivity. This study examined whether transformation of supramolecular assembly of root cell wall polysaccharides (CWPs) contributes to genotype-specific responses to Al stress in triticale. CWPs were extracted from apical and hairy root segments of two triticale genotypes, differing in Al tolerance. Water-extractable polysaccharides (WEPs) and those extracted with trans-1,2-cyclohexanediaminetetraacetic acid (CDTA) and sodium carbonate (Na_2_CO_3_) were analyzed using the multi-detection high-performance size-exclusion chromatography (HPSEC-RI-LALS/RALS-DV-UV-Vis). WEPs most clearly reflected differences between genotypes in macromolecular organization and Al-induced modification. Both root segments contained high molar mass (HM) subunits of WEPs with distinct anisotropic light scatterer (AS) domains. AS domains of a tolerant genotype were symmetrically elongated and branched, whereas those of a sensitive one were asymmetrically elongated with a spherical shape. In both genotypes, Al stress induced an association of apical HM subunits to higher molar mass forms, but in a different manner. The tolerant genotype maintained branched AS domain architecture by forming separate HM subunits that prevented Al infiltration. In contrast, the sensitive genotype showed complete merging of all HM subunits into a micro gel structure, leading to AS surface degradation. These findings provide novel insight into the role of root AS domains and supramolecular cell wall organization in plant adaptation to abiotic stress.

## 1. Introduction

Acidic soils, representing about 50% of agricultural lands in the world, are recognized as one of the major factors limiting crop productivity, in particular, when soil pH drops below 5.0 [1,2]. This is ascribed to the transformation of non-toxic aluminum (Al) silicates and oxides present in soil to soluble monomeric forms, among which the trivalent cations (Al^3+^) have the most negative effects on plant growth and productivity [1,3].

The main target of Al toxicity is the root apex, with the distal transition zone between the apical meristem and elongation zone being the most sensitive part of the root system that responds rapidly to micromolar concentration of Al cations [4,5,6]. The toxic effects of Al cations are associated with disruptions in major metabolic and physiological processes, starting from the rapid inhibition of root growth and development, which causes reduced water and nutrient uptake, impaired photosynthesis, oxidative stress, and damage of cellular organelles, as well as cytoskeleton disruption and programmed cell death [1,7,8,9].

The most documented mechanism of Al tolerance is its external exclusion based on the Al cations’ binding ability of deprotonated anions of organic acids, secreted by roots, such as citrate, maleate, and oxalate [1,10,11,12]. Roots can release other Al chelating substances, such as benzoxazinoids and phenolics [13,14]. Furthermore, the secretion of gel-like mucilages by roots and the formation of border cells may perform a similar function [15].

On the other hand, interaction between Al cations and the root cell wall is considered a key aspect of several internal tolerance mechanisms, which are induced in response to Al stress at various levels of the plant defense system. The first symptom is a modification of the cell wall that that causes changes in its integrity, physicochemical properties, and functionality [16].

The cell wall is composed primarily of polysaccharides with small amounts of proteins and phenolics. Cell wall polysaccharides (CWPs) are categorized as cellulose which forms a microfibrillar skeleton, and matrix polysaccharides, hemicelluloses, and pectin, which interact with the surface of cellulosic microfibrils and fill the spaces between them. Matrix polysaccharides are highly complex polymers, composed of an array of heterogeneous subunits with self-assembly characteristics [17,18].

In grass primary walls (type II), highly branched glucuronoarabinoxylan (GAX) and unbranched (1→3), (1→4)-β-D-glucan (BG) predominate, whereas pectin and xyloglucan (XyG) are minor components, in contrast to the pectin- and XyG-rich cell wall of dicots (type I) [17,19]. In addition to GAX and BG, other polysaccharides, such as arabinogalactans (AG), arabinans, galactans, and XyG, were found in the cell wall fractions of wheat roots [20,21]. The most abundant polysaccharide is GAX, with a backbone composed of 1→4-linked β-D-xylopyranosyl (Xyl*p*) residues. Its xylan chains are partially substituted at the *O*-2, *O*-3, and/or both *O*-2 and *O*-3 positions with α-L-arabinofuranosyl (Ara*f*) residues. Ara*f* units are, to a certain extent, ester-linked with phenolic acids residues, mostly with ferulic acid (FA) at the *O*-5 position [22]. Ara*f* can form short oligomeric branches of the xylan chains [23]. Xylan backbones also bear other substituents, such as *α*-D-glucuronic acid (GlcA) and its 4-*O*-methyl derivative residues at the *O*-2 position, and acetyl residues at the *O*-2 and *O*-3 of Xyl*p*. The structural heterogeneity of GAX is mainly ascribed to its variation in the degree of substitution with different substituents, different proportions of substituted and unsubstituted Xyl*p* residues that occur in the backbone, as well as diversity in its molar mass [23].

The mechanism of Al tolerance driven by electrostatic interactions, where Al cations bind to negatively charged cell wall components, such as pectin domains with linear 1→4-linked *α*-D-galacturonic acid (GalA) residues and side GlcA residues of xylan chains, is well known [4,24]. Other hemicelluloses that do not exhibit cation exchange ability, such as xyloglucans (XyG), have also been shown to participate in Al stress mitigation. Zhu et al. [25] showed that reduced O-acetylation of XyG increases hydroxyl group availability, enhancing Al binding in the cell wall. This points to a subtle yet important role of hemicelluloses in modulating wall properties that affect Al accumulation. Modification of cell wall polysaccharide composition in response to Al cations are shown to be crucial for enhancing Al tolerance in wheat, barley, rice, triticale, and Arabidopsis [26,27,28,29,30]. However, their spatial distribution, ultrastructure, and supramolecular organization remain poorly understood.

Root water-extractable cell wall polysaccharides (WEPs), due to the presence of numerous hydroxyl groups influencing their hydrophilicity, exhibit strong water affinity, promoting wall swelling, hydration, and structural flexibility. They form the outermost dynamic layer, which is the first barrier to face stressful conditions with the potential to act as a sensors and/or buffer. Hence, the study of root WEPs offers a valuable approach for explaining the role of these polysaccharides in plant Al tolerance mechanisms. In our previous work [31], we have developed an imaging multi-detection high-performance size-exclusion chromatography (HPSEC) method that allows us to track changes in the macromolecular characteristics of individual subunits present in a complex matrix of WEPs after specific enzymatic digestion of a single or a few polysaccharide components. This method can serve as a useful tool in the observation of the supramolecular assembly of cell wall polysaccharides in different plant tissues, including the root cell wall matrix in stress adaptation.

Here, we investigate whether WEPs, as the outermost and most responsive cell wall fraction, exhibits structural signatures in triticale root segments that are related to Al tolerance. The study aimed to characterize the supramolecular structure of WEPs isolated from roots of two triticale genotypes contrasting in Al tolerance, focusing on different root regions, apical and hairy root zones, under Al stress. By employing physicochemical and imaging multi-detection HPSEC techniques, we tested the hypothesis that Al stress induces specific macromolecular organization of WEPs, particularly in tolerant genotypes, which may reflect active defense mechanisms. We demonstrated the occurrence of unique high molar mass structures with the ability to scatter light anisotropically in WEPs of both root segments. Furthermore, we elucidated the mechanisms of stress-induced transformation of supramolecular assembly and their contribution to Al tolerance of triticale.

## 2. Results

### 2.1. Changes in the Amount of Extracted Fractions and the Composition of Matrix Polysaccharides as a Response to Aluminum Ion Stress

#### 2.1.1. Yield of Isolated Fractions

**Figure 1:**
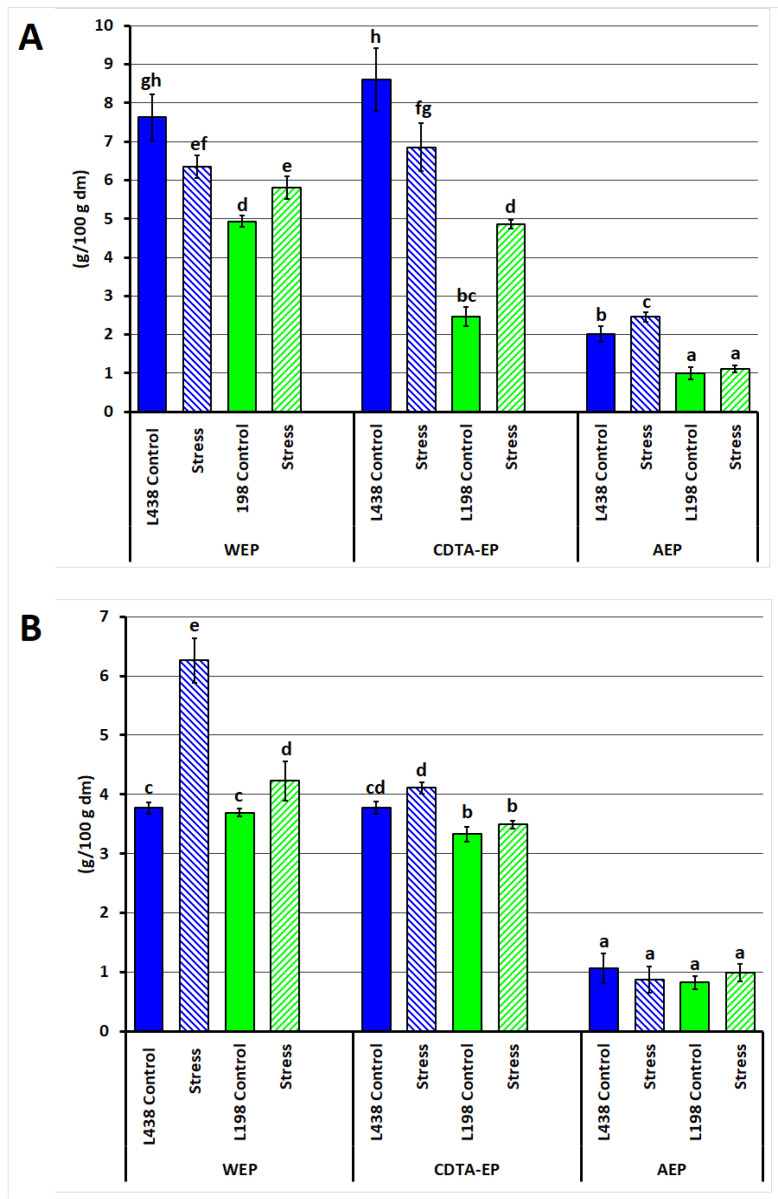
Yield (% of starting root sample, and dry matter, dm) of water-, CDTA- and alkali-extractable cell wall polysaccharide fractions obtained from (**A**) apical segments and (**B**) hairy root samples of the Al-sensitive (L438) and the Al-tolerant (L198) triticale genotypes under control and Al stress conditions. Data are represented by the mean ± SD of three replicates. Different letters indicate significantly different values (*p* < 0.05).

External, weakly bound cell wall (CW) fractions were isolated from the apical and hairy root segments of two triticale lines (sensitive and tolerant to Al^3+^) by sequential extraction with water, CDTA, and weak alkali, designated as water-, CDTA- and alkali-extractable polysaccharides (WEPs, CDTA-EPs, and AEPs, respectively). Their sum (WEPs + CDTA-EPs + AEPs) in the CW of the apex was 18.3% for the sensitive line and 8.4% for the tolerant line (Figure 1A). Under Al^3+^ exposure, the WEP and CDTA-EP content in the apices of the sensitive line decreased by ~20%, while in the tolerant line, WEPs increased by 15%, and CDTA-EPs doubled. In hairy roots, Al stress induced a more than twofold increase in WEPs and a 10% increase in CDTA-EPs in the sensitive line, with a weaker tolerant line response (~8% increase in WEPs) (Figure 1B).

#### 2.1.2. Composition of the Fractions

Because plant CW is composed mainly of polysaccharides, we assessed the content and composition of matrix polysaccharides to determine whether there are some clear differences between the CW fractions isolated as well as between both root segments. The varied content of polysaccharides along with uronic acids (UAs) was observed in the extracted fractions (Table 1). WEPs contained the highest amounts of neutral matrix polysaccharides and UAs: 22.5 and 32.9% in the apices and 34.6 and 32.2% in the hairy root segments of the sensitive and tolerant lines, respectively, in contrast to AEP fractions containing approximately 15% of neutral sugars and UAs in the apices and 15.4/9.1% in the hairy root segments (sensitive/tolerant). The CDTA-EPs contained 10.9/13.9% and 6.1/7.0%, respectively. Dominant monosaccharides were arabinose (Ara), galactose (Gal), xylose (Xyl), and glucose (Glc), with smaller quantities of mannose (Man), rhamnose (Rha), and fucose (Fuc). In WEPs, Ara and Gal constituted ~25% of polysaccharide fraction, each: Xyl: 15.1/18.8% in apices and 11.2/19.1% in hairy root segments. CDTA-EPs and AEPs contained mainly Ara and Xyl (20–30%). Glc in WEPs was 16.2–23.8% in apices, half as much than in hairy root segments. The composition of neutral sugars changed slightly in CW fractions extracted from both root segments of sensitive and tolerant lines and upon the stress. The highest UA content was found in CDTA-EPs of hairy roots (13.2/11.8%, sensitive/tolerant line), less in apices (4.9 and 7.3%, respectively); upon the stress, the UA content in apices increased more than double, with no changes in hairy roots. The increase in UA content was also accompanied by a significant increase in the proportion of UA/Rha (from 0.91 to 3.1 for the sensitive line and from 1.4 to 5.3 for the tolerant one).

### 2.2. Solid State NMR Characterization of WEPs from Root Apex Exposed to Al Stress

**Figure 2:**
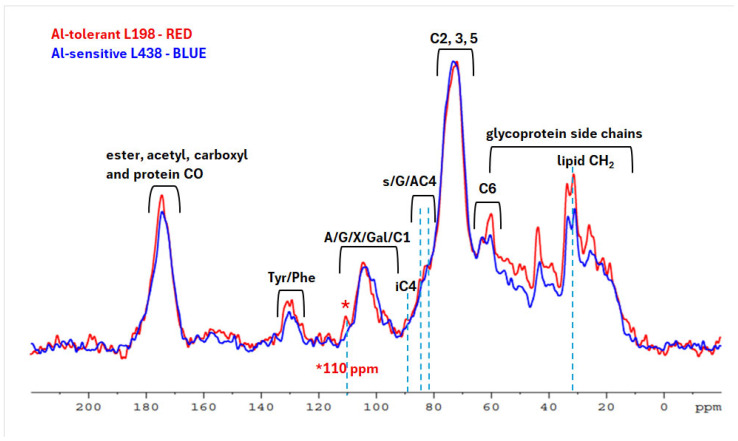
^13^C CP/MAS NMR spectra of WEP fractions isolated from root apices of the Al-sensitive (L438) and the Al-tolerant (L198) triticale genotypes after Al treatment. For interpretation of the references to color in this figure legend, the reader is referred to the web version of this article.

Next, we aimed to establish if solid-state ^13^C NMR would provide more obvious indications for detection of WEP constituents that differentiate both triticale. The ^13^C CP-MAS NMR spectra of WEP fractions, isolated from root apices after treatment with Al cations, generally showed minor changes in the splitting patterns and intensity between corresponding signals of carbon resonances, which pointed to the differences in their fine structure (Figure 2). The signals seen at 72–78 ppm, which originate from C-2, C-3, and C-5 of glycans, were predominant. Other prominent overlapped peaks with a clear maximum at 174 ppm in the carbonyl region (170–180 ppm) indicated the presence of negatively charged COO^–^ (176 ppm), acetyl (OCOCH_3_, 174 ppm), and methyl esters (COOCH_3_, 172 ppm) [32].

Numerous protein resonances in the range of 10–60 ppm included signals from glycine (43.5 ppm), proline (25.7; 60.0 ppm), tyrosine (130; 157 ppm), and phenylalanine (129 ppm) [33], which were stronger in the tolerant line. Methyl ester signals (53 ppm) and those of acetyl ester groups (21 ppm) were hardly noticeable since they overlapped with protein peaks. Spectra of both lines showed a lipid CH_2_ doublet at 33 and 31 ppm, stronger in the tolerant line.

The key difference was the C-4 peak of the interior crystalline cellulose (iC) at 89 ppm in WEP fraction, predominant in the tolerant line, indicating the presence of cellulose microfibrils in the WEP fraction [34,35,36]. The iC signal was accompanied by C-4 signals at 82–85 ppm, where 81.7 ppm corresponds to C-4 xylan bound to cellulose fibrils, and 83.5 ppm characterize that unbound structure. All those signals are stronger in the tolerant line. Another fundamental difference is a 110 ppm peak ascribed to C-1 5-Ara, 3,5-Ara, 2,3,5-Ara, and T-Ara of highly branched (1→5)-α-L-arabinan [37,38,39], observed only in the tolerant line.

### 2.3. Identification of Different Subunits in Matrix CWPs and Their Macromolecular Characterization

#### 2.3.1. Polysaccharide Subunits with Different Molar Mass

**Figure 3:**
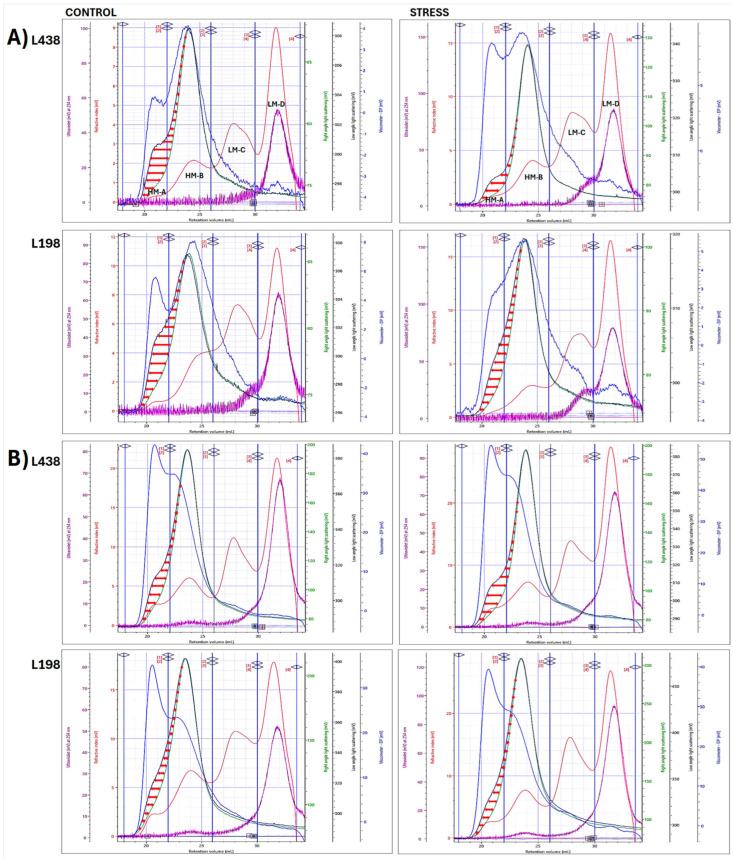
Superimposed multi-detector HPSEC chromatograms of **WEP** fractions isolated from (**A**) apical segments and (**B**) hairy root samples of the Al-sensitive (L438) and the Al-tolerant (L198) triticale genotypes as detected by five detectors: RI (**red line**), DV (**blue line**), RALS (**green line**), LALS (**black line**), and UV254 nm (**purple line**). The horizontal red arrows (**↔**) indicate the discrepancies between the signals of the two light scattering detectors, LALS and RALS, for LS 7° and 90°, respectively. The signals of all detectors have been scaled to improve visualization. For interpretation of the references to color in this figure legend, the reader is referred to the web version of this article.

To test if macromolecular characterization of CWP fractions can bring some important observations, which may help answer the question why only the tolerant line shows root regrowth ability after Al stress, we used the HPSEC system with four detectors in a line. The analysis of WEP fractions showed the presence of four subunits (the RI-chromatograms, red lines) in root apices (Figure 3A) and hairy root segments (Figure 3B). The high molar mass (HM) subunits, HM-A and HM-B, were indicated by strong signals of low- and right-angle light scattering (LALS and RALS) detectors, measured at 7° and 90° (black and green lines, respectively), and accompanied by a strong signal of viscometer (DP) (blue line). Of the two low molar mass (LM) subunits, LM-C and LM-D, the latter one that eluted in the lowest molar mass region (30–34 mL), showed a UV signal that followed the RI detector signal. The UV signals recorded at two wavelengths, 254 and 325 nm, the in the HM region (22–26 mL), indicated the presence of phenolic acids in the HM-B subunit extracted by Na_2_CO_3_ from apex and those extracted by water and Na_2_CO_3_ from hairy roots (Figures S1 and S2) [40]. The UV254 nm signals in the LM-C region (26–31 mL) were assigned to AG-P [31], whereas those detected in the LM-D (31–34 mL) pointed to the presence of CW phenolics; however, proteinaceous substances cannot be ruled out.

#### 2.3.2. Identification of AS Domain

There was a significant discrepancy between LALS and RALS detector signals that pictured the convex lens-like surface (marked with red horizontal arrows) in the macromolecules with the highest weight average molecular weight (*M*_w_) (18–24 mL), unlike the remaining HM counterparts (24–26 mL) with the same signals of both LS detectors (Figure 3). These molecules displayed high angular dependance of the scattered light intensity, as RALS signal was shifted towards lower *M*_w_, in comparison to that of LALS detector, which measures the scattered light at a very low angle; thus, the angular effects are negligible.

Very clear differences in the shape of the apical AS surface existed between the two triticale lines, i.e., the asymmetrically and symmetrically elongated AS domains were found in the sensitive and tolerant triticale lines, respectively (Figure 3A). Similar differences, but much less pronounced, were found in hairy root segments (Figure 3B). Their counterparts from CDTA-EP and AEP fractions (Figures S3 and S4), also having a significant size (Tables S1 and S2), showed very little or almost no angular dependence.

#### 2.3.3. Yield and Macromolecular Characterization of Subunits

Of the four WEP subunits (Figure 3), the HM ones accounted for 19.5 and 21.5% in the apex and 23.3 and 26.4% in hairy root segments, for the sensitive and tolerant lines, respectively (Table 2). The minor HM subunit, HM-A, amounted to 15.4 and 12.1% of the entire HM-subpopulation in root apical segments and 25.3 and 20.8% in those of hairy roots. This monodispersed subunit (*M*_w_/*M*_n_~1.00) was distinguished by very high values of intrinsic viscosity [*η*] and large radii (>100 nm).

The structure sensitive parameter expressed by ratio of radius of gyration to hydrodynamic radius (*R*_g_/*R*_h_) is determined by shape, size, and dispersity (*M*_w_/*M*_n_) of macromolecule in a solution, but independent of its molar mass. Its values (0.79 and 1.37, respectively) showed that an apical HM-B subunit of the sensitive line displayed a spherical conformation in dilute solution, in contrast to branched macromolecule found in the tolerant line (Table 2) [41,42]. On the other hand, the Mark–Houwink exponent a (M-H a) values (0.86 and 0.54, respectively), which depend on the polymer–solvent system and temperature, but also on molar mass, indicated that the dominating HM-B subunit of the sensitive line adopted more extended conformation with semi-flexible chains, in comparison to a random coil shape with flexible chains of the tolerant one.

By contrast, the dominating HM-B subunits of hairy root segments showed micro gel conformation (*R*_g_/*R*_h_, 0.62 and 0.66) [43] with semi-flexible and flexile chains (M-H a, 1.06 and 0.75, for the sensitive and tolerant lines, respectively). The values of *R*_g_/*R*_h_ ratio obtained for minor HM-A subunits indicated that only that of the tolerant line had micro gel conformation, whereas non-spherical, elongated shape was observed in the sensitive one (*R*_g_/*R*_h_, 0.60 and 0.93, respectively). The minor HM-A subunit from the tolerant line was characterized by the highest *M*_w_, which was markedly higher that of the sensitive one. However, there were no significant differences between lines in *M*_w_ of dominating HM-B subunits. Unlike the apical HM subunits, those of hairy root segments were feruloylated, as indicated by UV detector (Figure 3B), and a two-times-higher feruloylation degree was found in the tolerant line (Table 2). Moreover, both HM subunits of hairy root samples exhibited a significantly higher *M*_w_ compared to their counterparts from apical segments.

### 2.4. Changes in Macromolecular Characteristics of High Molar Mass Subunits Caused by Al Stress

#### 2.4.1. Al-Induced Transformation of AS Domain Profile

Under stress, the apical AS domain of the sensitive line, enclosed by LALS and RALS signals (red vertical arrows) (Figure 3A), was significantly reduced, but in a specific way, i.e., the biggest changes concerned its longitudinal dimension. In the tolerant line, the changes were much smaller. They were related exclusively to a small reduction in its transverse diameter, whereas there was no change in its length. Furthermore, there was a clear change in the RI profile (red line) of minor HM-A subunits, observed only in the tolerant line. This subunit was no longer a separate population, but it became an integral part of the dominating HM-B subunit.

Subtle changes, caused by Al stress, were observed in the hairy root AS domains (Figure 3B), as their initial forms were much narrower, resembling very flat convex lenses. Small changes in AS domain of the tolerant line were related only to the reduction in its width, while for the sensitive one, a partial reduction in its length with increase in the area at the bottom of the LS peak (front arm) were noted.

#### 2.4.2. Al-Induced Transformation of Macromolecular Parameters

In the case of apical HM-B subunits, Al stress induced twofold and threefold increase in *M*_w_ of the sensitive and tolerant lines, respectively; nevertheless, both reached a similar values of *M*_w_ (Table 2). It was coupled with a reduction in dispersity (change in *M*_w_/*M*_n_ from 1.49 to 1.29) only of the tolerant line. Although Al stress resulted in lower *R*_g_/*R*_h_ values of all HM subunits, those from apical segments of the tolerant line retained their branched conformation (*R*_g_/*R*_h_, 1.18 and 1.04, respectively for HM-A and HM-B) and chains flexibility of dominating the HM-B subunit, as shown by an M-H value of 0.61. In contrast, the dominating HM-B subunit of the sensitive line adopted a micro gel conformation (*R*_g_/*R*_h_ < 0.70) and retained semi-flexible chains (MH a, 0.83). The minor HM-A subunit, however, was transformed from a branched molecule to a slightly extended spherical conformation (*R*_g_/*R*_h_ = 0.83).

Al stress caused a significant decrease in *M*_w_ of the hairy root HM subunits of the sensitive line and substantial increase in *M*_w_ of those coming from the tolerant line (Table 2). It was accompanied by, again, a notable decrease in HM-B subunit dispersity of the tolerant line. The *R*_g_/*R*_h_ ratios showed that micro gel conformation of the HM-B subunit of the sensitive line turned into a spherical one (*R*_g_/*R*_h_ = 0.70) with increased stiffness of its semi-rigid chains (change in MH a from 1.06 to 1.21). The micro gel conformation of HM-B subunit of the tolerant line, conversely, was preserved under stress conditions, with increased stiffness of its chains from flexible to semi-flexible ones (change in MH a from 0.75 to 0.80).

Further CW fractions, CDTA-EP and AEP, were dominated by proteoglycans concentrated in the LM-D subunits, Figures S4 and S5), which amounted to 78–88% of the total amount of material recovered after extraction (Tables S1 and S2). Thus, the HM subunits constituted only 5.1–6.5% in CDTA-EPs and 8.2–17.2% in AEPs. In general, CDTA-EP polymers showed higher *M*_w_ than those of WEP counterparts and the highest *M*_w_ was found in AEP fraction. The stress-induced changes in their *M*_w_ and conformation and chain stiffness, indicated by *R*_g_/*R*_h_ and M-H a values, were similar to those observed in the WEP fraction.

### 2.5. Effect of Al Stress on Supramolecular Assembly of High Molar Mass WEPs

**Figure 4:**
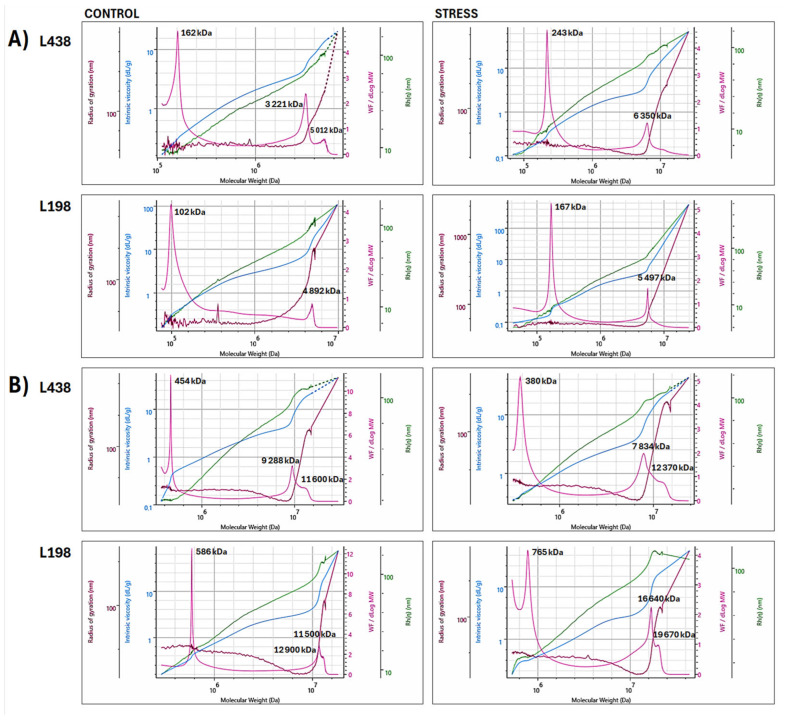
Superimposed bi-log plots of macromolecular parameters (*M*_w_, *R*_g_, *R*_h_, and [*η*]) versus *M*_w_ of **WEP** fractions isolated from (**A**) apical segments and (**B**) hairy root samples of the Al-sensitive (L438) and the Al-tolerant (L198) triticale genotypes, obtained for high and low molar mass (**HM** and **LM)** regions in the range of retention volume of 18–34 mL (*M*_w_–**pink line**, *R*_g_–**brown line**, *R*_h_–**green line**, and [*η*]–**blue line**). For interpretation of the references to color in this figure legend, the reader is referred to the web version of this article.

**Figure 5:**
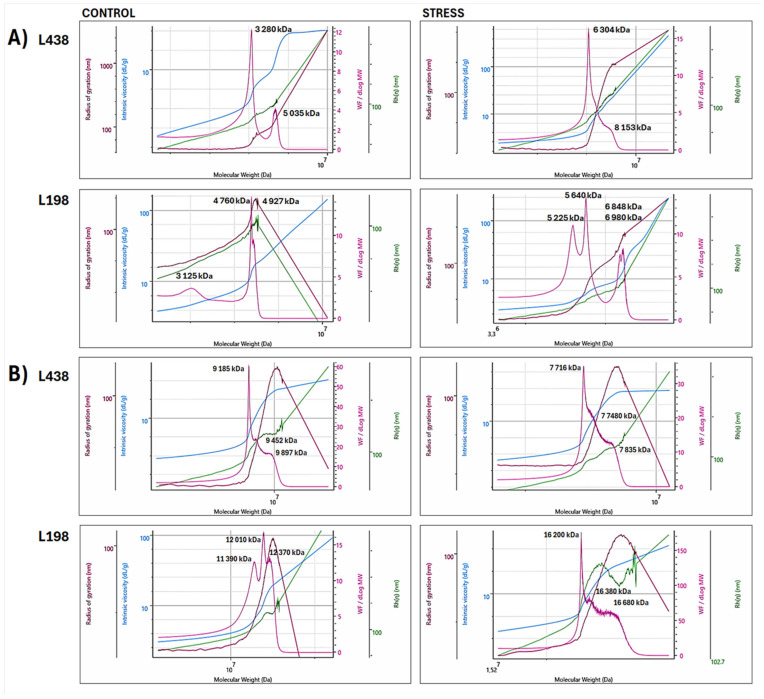
Superimposed bi-log plots of macromolecular parameters (*M*_w_, *R*_g_, *R*_h_, and [*η*]) versus *M*_w_ of **WEP** fractions isolated from (**A**) apical segments and (**B**) hairy root samples of the Al-sensitive (L438) and the Al-tolerant (L198) triticale genotypes, obtained for high molar mass (**HM**) regions in the range of retention volume of 18–26 mL (*M*_w_–**pink line**, *R*_g_–**brown line**, *R*_h_–**green line**, and [*η*]–**blue line**). For interpretation of the references to color in this figure legend, the reader is referred to the web version of this article.

Because the changes in the conformation of AS domains induced by Al stress revealed the general mechanisms of their transformation, we employed the superimposed distribution of all macromolecular parameters to take insight into their supramolecular organization and understand the basis of these mechanisms. The superimposed bi-log plots of four macromolecular parameters (*M*_w_, *R*_g_, *R*_h_, and [*η*]) versus *M*_w_ of the WEP fractions (Figure 4), obtained for both HM and LW regions, showed that HM populations significantly affect the conformation of the entire molecule, seen as changes in the slope of a curve in the Mark–Houwink plot (blue line), despite the fact that the HM populations are smaller than those with LM. The detailed molar mass and radii distribution and conformation changes obtained only for the HM region (retention volume of 18–26 mL) are shown in Figure 5. In this region, there were substantial differences in molar mass distribution between both triticale lines of apical segments as well as those of hairy roots, which were already observed in their initial forms.

In the case of apical segments, the tolerant line contained two separate HM populations, a relatively small one with a peak at 3125 kDa and a prevailing one centered at 4760–4927 kDa (Figure 5A). The latter one is very unique, as it strongly bends both radii (*R*_g_ and *R*_h_ peaks). The Al stress induced their specific association, which resulted in two separate doublets at 5225 and 5640 kDa and 6848 and 6980 kDa. The sensitive line also contained two separate populations in the HM region, the dominating one with a peak at 3280 kDa and the smaller one with a peak at 5035 kDa. Unlike the tolerant line, Al stress caused their association to be a single very broad population ranging from 6304 to 8153 kDa.

The hairy root segments of triticale lines, in contrast to their apical segments, contained a single HM population built of at least three associated subunits with peak *M*_w_ ranging from 9185 to 9897 kDa in the sensitive line, and from 11,390 to 12,370 kDa in the tolerant one (Figure 5B). In the case of the tolerant line, Al stress caused association of initial HM population with high molar mass form, again, made of at least three interconnected subunits with *M*_w_ ranging from 16,200 to 16,680 kDa. The initial HM population of the sensitive genotype, in contrast to that of the tolerant line, was depolymerized to a form with a significantly reduced molar mass (7716–7835 kDa).

## 3. Discussion

### 3.1. Basic Characteristics of Root Cell Wall Fractions Isolated by a Sequential Extraction

The yield of root CW fractions released during consecutive extraction was relatively low, as mild extractants were intentionally used, to collect the outermost fractions, being spread on the CW surface and/or weakly bound to a core inner structure. Such extraction minimalizes the risk of alteration and degradation of material isolated, which must be taken into account in the case of strong extractants, effectively increasing the extraction yield of CW fractions. The data indicated that the outer layer analyzed in the root apex of the tolerant line is much smaller than that of sensitive one; however, it is enriched in WEP components (Figure 1). Unlike the sensitive line, the tolerant one triggers the synthesis of the two dominant fractions under stress conditions.

The compositions of matrix polysaccharides present in all three CW fractions, sequentially extracted from apices and hairy roots, were similar (Table 1), which is consistent with previous reports on root CWPs, extracted by a comparable procedure from wheat [20,21]. This indicates that all fractions were a very complex mixture of many analogous polysaccharides. The WEP fractions were enriched in both Ara and Gal, unlike CDTA-EP and AEP ones, in which Ara dominated, pointing to the presence of arabinans, galactans, and AG-P. However, some amounts of Ara residues can derive from side substituents of xylans and pectin, and those of Gal from XyG and pectin [44,45].

Quite the opposite, the lowest levels of Xyl residues were observed in WEP fractions from apices and hairy roots, while their increasing amounts were found in subsequent extracts, indicating much stronger associations between noncellulosic polysaccharides present in these fractions and cellulose, which can be mediated by xylan with known affinity to cellulose [46,47]. This was also observed in a previous report [20] and is supported by the fact that lignocellulosic residue (RES), left after sequential extraction of CWPs, contained ~50% of Xyl residues, confirming that xylan was a major matrix polysaccharide in the CW of triticale roots. However, a small amount of Xyl units may also come from XyG, which is a minor component of grass CW [17].

The presence of Glc-containing polysaccharides, such as mixed-linkage (1→3)(1→4)-β-D-glucan and (1→3)-β-D-glucan (callose), has previously been demonstrated in wheat roots [20,21]. Furthermore, small amounts of Glc can also come from XyG and glucomannans.

As could be expected in the case of cereal cell walls, UAs constituted a relatively minor amount in all CWP fractions, indicating the presence of pectins. Their highest contents were found in CDTA-EP fractions isolated from hairy root segments, while their analogues from apices had almost two-times-less uronic acids. Despite the highest UA content in the CDTA-EP fractions of hairy roots, Al stress did not cause any significant changes in their level, whereas a substantial increase in UA content, induced by Al stress, was observed exclusively in the case of CDTA-EPs from apices with significant increase in the proportion of UA/Rha, indicating a simultaneous increase in the smooth homogalacturonan (HG) regions of pectin, especially pronounced for the tolerant line. This suggests that only pectin present in this fraction had free carboxylic residues with the ability to chelate cations, which responded to Al stress, in contrast to methylated analogues present in the remaining fractions. The occurrence of a small amount of pectin in each CWP fraction, regardless of the type of solution used for extraction, is in line with the latest reports, which evidenced covalent linkages between pectin and xylan as well as between pectin, xylan, and AG-P, pointing to cross-linking role of pectin in the polysaccharide matrix of CW [39,48].

The ^13^C NMR spectra of apical WEPs exposed to Al stress showed that in the tolerant line, they were much strongly associated with cellulose microfibrils (Figure 2). The cellulose resonances have been previously detected in the ^13^C CP-MAS NMR spectra of WEP fractions isolated from oat grain cell walls, showing that even matrix polysaccharides that are easily extractable with water contain hydrophobic cellulose microfibrils [31]. Another fundamental difference between both spectra was the presence of a small subunit of highly branched (1→5)-α-L-arabinan composed of contiguous branched units exclusively in the tolerant line [37]. Although the ^13^C NMR revealed only small structural differences between both lines in the apical WEP structure, they could be important for cell wall functionality, since it is known that relatively small structural differences may cause significant changes in physicochemical properties of CWPs [49].

### 3.2. Identification of Anisotropic Scatterer Domains and Their Macromolecular Characterization

Although the CWPs of plant roots have been intensively studied by commonly used in situ methods, such as monoclonal antibodies and multidimensional ^13^C correlation solid-state NMR, there is a lack of research on macromolecular properties of these polysaccharides, complementing the data obtained from in situ or other methods [36,50,51,52]. In this study, apart from the basic macromolecular characteristics of CWPs investigated, which allowed us to draw conclusions about the general mechanism of their transformation during Al stress, we provided detailed insights in their supramolecular organization and its modifications induced by Al stress. This is the basis for our understanding of the general mechanisms revealed by macromolecular parameters of HM subunits. The multi-HPSEC imaging method for investigation of supramolecular assembly of CWPs was recently used in our previous study on xylan-cellulose interactions in oat, as we introduced a sequential scanning of very small subunits of the macromolecule by applying the multi-HPSEC system [31].

**Figure 6:**
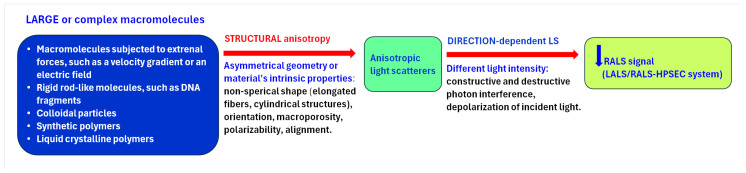
Effect of structural anisotropy of macromolecules on right-angle light scattering (RALS) detector signal.

The multi-detection HPSEC analysis enabled preliminary categorization of root CWPs present in each fraction into polysaccharides, feruloylated polysaccharides, glycoprotein, or proteoglycan with different molar mass, (Figure 3 and Figures S1–S4). The data obtained by light scattering detectors, LALS and RALS, evidenced the presence of AS domains, exclusively in the HM region of the WEP fractions from both apical and hairy root segments (Figure 3). They are composed of complex polysaccharides that are not conjugated with proteins. It is commonly known that owing to their structural anisotropy, such domains present in large or complex macromolecules show angular dependence of light scattering, i.e., they scatter light in different directions with different intensities. This causes partial destructive interference of photons, which are depolarized, and thus negatively affects the intensity of the RALS detector signal, measured at 90° (Figure 6). In contrast, the intensity of LALS detector signal, measured at an angle of 7°, is practically unaffected by angular dependence.

The apical AS domains are not feruloylated, while the hairy root analogues contain a small amount of feruloylated chains. The shape of AS domains, reflecting interaction space between the two HM subunits, the minor HM-A and predominant HM-B, clearly distinguishes the AS domain present in apical segments of the tolerant genotype from that of the sensitive one (Figure 3). In hairy root segments, similar changes, but much less pronounced, are observed. The symmetrically elongated shape of AS domains observed in the tolerant genotype and those asymmetrically elongated of the sensitive one, indicated the importance of the symmetry in the structure of specific outermost root tissues. This may be related to the presence of small structural elements, among others, such as highly branched arabinan subunits, and to stronger interaction with cellulose microfibrils, as indicated by solid-state ^13^C NMR analysis (Figure 2).

The structural parameter *R*_g_/*R*_h_ indicated that dominant HM-B subunits of WEPs obtained from apical segments displayed branched conformation in the tolerant line, while that of the sensitive one had a distinctly different spherical shape (Table 2). This structural parameter might be used as an index of Al tolerance in screening of triticale breeding populations. Nevertheless, it is very possible that similar dependencies may also occur in triticale parental species, wheat and rye. The most important advantage of this parameter is its ability to indicate a micro gel structure among other conformations of the polymers.

Moreover, the Mark–Houwink exponent ‘a’ is a valuable tool for the assignment of conformation type with conformation flexibility for many polymers in good solvents. The values of M-H a parameter summarized in Table 2, Tables S1 and S2, clearly indicated that dominating HM-B is the most flexible one in each cell wall fraction released from apical segments with water, CDTA and Na_2_CO_3_, respectively. This suggests that these subunits could be the most suitable for growth, as it is known that the stiffest tissue limits organ growth [53]. On the contrary, all proteinaceous LM subunits exhibited semi-flexible conformation with different degrees of stiffness. The pioneering research established that flexible macromolecules become deformed on flow, and thus exhibit optical anisotropy [54]. In addition to high flexibility, HM-B subunits exhibited random coil conformation in solution, which contains much more spaces between the constituent chains, in comparison to the remaining conformational extremes, those with a compact sphere or more extended, rigid rod-like shapes. This is in line with the higher porosity of cell wall matrix, and implies easier flow of the solutes, ensuring their transport across the cell wall.

Interestingly, the minor HM-A subunit present in both root segments, especially those from WEPs, exhibited extremely high values of [*η*], illustrating the volume occupied by a single polymer chain, which were comparable to those of commercial guar and xanthan gums (1135 and 1355 mL g^–1^, respectively) [55] with well-known strong hydration properties as well as the ability to create highly viscous solutions at very low concentrations (Table 2). In addition, the HM-A subunit was characterized by large radii (~100 nm), whose values can be related to those reported earlier for aggregated macromolecules of AX and β-glucan from oat and wheat grains [56]. This indicates that HM-A subunits may have the ability to control the flow of LM solutes in the root cell wall. Although it occurs in small amounts, its specific location, i.e., in the front of the lens-like AS domain, may support this assumption. On the other hand, this subunit is eluting in the region of the highest molar mass (Figure 3). Our previous study demonstrated that in this region, matrix polysaccharides strongly interacted with surface cellulose microfibrils [31]. Such associations are also detected in this study by ^13^C NMR analysis, suggesting that this small HM-A subunit interacts with both surface cellulose microfibrils and dominating HM-B subunits.

### 3.3. Anisotropic Scatterer Domains Are Transformed Under Al Stress by Genotype-Specific Manners of Their HM Subunits Associations

This study showed that the preservation of AS domain of triticale root apex under Al stress, particularly its length, is a key factor, ensuring root regrowth after the stress (Figure 3A). In this particular case, the molar mass of associated polymers does not matter, but the way of association is important. This was ascribed to different mechanisms of association of HM polymers of apical segments during Al stress, observed in triticale genotypes with diverse sensitivity, as revealed by their changes in supramolecular assembly (Figure 5A). Different association manner of HM subunits induced by Al stress resulted from diametrically opposed initial conformation of these subunits, i.e., spherical and branched, as indicated by their values of *R*_g_/*R*_h_ (Table 2) and by differences in distribution of both radii versus *M*_w_ (Figure 5A).

The HM-A subunit with the highest *M*_w_ of the tolerant line showed very exceptional close-fitting association between its chains, pointed by huge overlaid *R*_g_ and *R*_h_ peaks, indicating very tight associations between polysaccharide chains [57]. This means that Al cations can be mainly excluded; however, they may interact only with particular regions of these HM subunits, in which the cell wall structure is weaker or/and there are a small domains of the unmethylated homogalacturonan region of pectin and/or GlcA residues of xylan chains. Consequently, Al stress induces formation of separated HM subunits in the apical segments of the tolerant genotype that maintains their branched structure and chain flexibility, saving the AS domain. Although Al stress substantially reduces the force of interactions between the chains of HM subunits (Figure 5, reduced *R*_g_ and *R*_h_ peaks), due to distinct construction of HM subunits, the spread of Al cations through the entire HM population is blocked. This is supported by the fact that a significantly higher Al content was found in root apex of the sensitive wheat genotype, in comparison to that of the tolerant one [58].

Unlike the initial HM subunits from apical segments of the tolerant line, those present in the sensitive one showed very weak association between their chains (very small *R*_g_ and *R*_h_ peaks); thus they were very loose in structure (Figure 5A). Such a structure can accumulate the toxic Al cations on the outermost layers of the subunit, which easily permeate to the inner cell wall structure. Hence, Al stress results in the formation of one completely integrated HM subunit, which was transformed towards a micro gel structure. It acts as adsorption gel for Al cations, drastically reducing interactions between HM subunit chains, leading to destruction of the AS domain. It is very likely that the left AS surface that eluted in the region of the highest *M*_w_ (Figure 3A) is strongly associated with cellulose microfibrils, as mentioned above; therefore, it resisted destruction. Nevertheless, its presence has no effect on the ability of the roots of the sensitive seedling to regrowth. This emphasizes the importance of maintaining longitudinal dimensions of AS domain under Al stress.

In the case of hairy root segments, the initial forms of dominating HM-B subunits of both triticale lines showed a micro gel structure, indicating their Al adsorption ability (Table 2). However, those of the tolerant line were characterized by much higher molar mass with a similar proportion of constituent subunits (Figure 5B) with a flexible random coil conformation of their chains (Table 2). The microstructure of the sensitive line was built of HM subunits with markedly lower molar mass enriched in forms with the lowest mass (Figure 5B). The Al stress resulted in association of HM polymers of the tolerant genotype to counterparts with higher molar mass and higher feruloylation degree. By contrast, stress-induced depolymerization of HM components was observed in the sensitive line. It is well-known that side residues of phenolic acids may cross-link polysaccharide chains, increasing a density of junction zones in a spatial polysaccharide network with strong hydration ability that is controlled also by polysaccharide size [59]. Hence, the considerable rise in molar mass and feruloylation degree of the HM polymers from hairy root segments of the tolerant line suggests their increased ability to adsorb Al cations, while decrease in molar mass of the sensitive genotype counterparts implies their lowered Al adsorption capacity, and much more Al ions can have a toxic effect on the root apex.

The optical response of AS domains, probably caused by deformation of their flexible chains during solute flow, indicates that these structures might be the part of flow channels in the outermost layer of triticale root. This is consistent with their random coli conformation, ensuring appropriate porosity of these subunits, and supported by their specific pattern in which the longitudinal dimension is the most decisive factor related to Al tolerance of triticale.

## 4. Materials and Methods

### 4.1. Plant Material

Seeds of two inbred lines of triticale (× *Triticosecale* Wittmack) with different tolerance to Al toxicity: the Al-tolerant line, L198 (*MAH3405 (Milewo*) × *Matejko*), and the Al-sensitive line, L438 (Gabo × 6944), were obtained from Plant Breeding Strzelce Ltd., Experimental Station Małyszyn, Poland. Both lines were characterized by high homozygosity (F10 generation) and were assessed annually for their level of Al tolerance, using the standard method according to Aniol [60], adapted by Niedziela et al. [61]. The genotypes used in this study have been previously described in terms of physiological and biochemical responses to Al stress by Niedziela et al. [61].

Briefly, seeds were surface-sterilized in 96% ethanol (1 min), followed by 1% sodium hypochlorite solution (15 min), and triple-rinsed with water (5, 10, and 15 min, successively). The seeds were germinated on a paper filter in a petri dish for 24 h at 20 °C in the dark until sprouting reached ~3 mm, and then sown onto polyethylene nets placed in trays, containing a base nutrient solution (2.0 mM CaCl_2_, 3.25 mM KNO_3_, 1.25 mM MgCl_2_, 0.5 mM (NH_4_)_2_SO_4_, 0.2 mM NH_4_NO_3_, and pH 4.5, EC 1.53). After 3 days of pre-treatment, seedlings were transferred for 24 h to the same medium supplemented with 16 ppm Al^3+^ (as AlCl_3_). Next, Al^3+^ ions were rinsed off, and seedlings were returned to the base solution for another 48 h to allow root regrowth. Roots of tolerant lines resumed growth, while those of sensitive lines did not.

Control plants were grown in an identical base medium without Al^3+^ supplementation. Experiments were carried out in a growth chamber (Pol-Eko Aparatura, ST500 B40 FOT10, Poland) under controlled conditions: 25 °C, 12 h photoperiod, and light intensity of 40 W·m^−2^.

Seedlings obtained after experiment were rinsed thoroughly with deionized water. Root apices (0–5 mn) and hairy root segments (roots fragments left after apex removal) were excised and immediately frozen at −80 °C, ground in liquid nitrogen using mortar and pestle, and then freeze-dried (Christ Alpha 1–4 lyophilizer, **Martin Christ, Osterode, Germany**). All measurements were based on at least four independent biological replicates.

### 4.2. Sequential Extraction and Purifications of Cell Wall Fractions

The apical and hairy root segments (100–150) mg were suspended with milli-Q water (1:20, *w*/*v*) in Pyrex glass tubes with screw caps and incubated with thermostable *α*-amylase (*Bacillus licheniformis*, 30 U, Megazyme, Neogen Corporation, Lansing, MI, USA) for 1 h in a boiling water bath with occasional shaking. The root samples were cooled to 40 °C, and incubated with amyloglucosidase (*Rhizopus* sp., 8 U, Megazyme, Neogen Corporation) and protease (*Bacillus licheniformis*, 40 µL, Megazyme, Neogen Corporation) at 40 °C for 16 h in a rotary shaker. The suspensions were centrifuged at 4500× *g* for 20 min in a Sigma 4–15 centrifuge (Sigma, Laborzentrifugen, Osterode, Germany). The supernatants were separated and the residues were suspended in 4 mL of milli-Q water, incubated at 40 °C for 2 h in a rotary shaker. The samples were centrifuged under the same conditions and the supernatants were combined and dialyzed with deionized water at 22 °C for 24 h using membranes with molecular weight cut-off of 12–14 kDa. The purified retentate was designated as WEPs. An aliquot (1.0 mL) of the retentate was immediately analyzed by multi-detection HPSEC and the rest of it was freeze-dried (Figure S5).

The residue left after water extraction was subjected to consecutive extraction with *trans*-1,2-cyclohexanediaminetetraacetic acid (CDTA) and sodium carbonate (Na_2_CO_3_), using a procedure adapted from Siedlecka et al. [62] and Müller-Maatsch et al. [63], respectively. The water-unextractable residue was suspended with 50 mM Tris-HCl (4 mL, pH 7.2), containing 50 mM CDTA, vortexed and incubated at 95 °C for 20 min. The slurry was centrifuged at 4500× *g* for 20 min. The supernatant was collected. The obtained residue was treated again with CDTA-containing buffer (95 °C, 20 min), centrifuged, and both supernatants were combined. The solution was dialyzed with deionized water at 22 °C for 24 h. The retentate was designated as CDTA-extractable polysaccharides (CDTA-EPs). It was immediately analyzed by HPSEC (1 mL) and the left retentate was freeze-dried.

The pellet left after CDTA extraction was mixed with 4 mL of 50 mM Na_2_CO_3_ solution, containing 20 mM NaBH_4_, and incubated overnight at 6 °C, using a rotary shaker. The supernatant was separated by centrifugation (4500× *g*, 20 min), and the extraction was repeated again (2 h, 6 °C). The combined supernatant was neutralized with acetic acid and dialyzed with deionized water at 22 °C for 24 h, and the obtained retentate was designated as alkali-extractable polysaccharides (AEPs). As in the case of WEPs and CDTA-EPs, it was immediately analyzed by HPSEC (1 mL) and freeze-dried.

### 4.3. Analytical Methods

The neutral sugar composition and content of non-cellulosic polysaccharides were analyzed by gas chromatography. The freeze-dried root CW fractions (10–15 mg) were hydrolyzed in 1 M sulfuric acid (100 °C, 2 h). The released monosaccharides were derivatized to alditol acetates, using a method described by Englyst and Cummings [64], and quantified in a gas chromatography system, which consisted of the Agilent 7890A series GC Custom chromatograph, equipped with a flame ionization detector and a DB-23 capillary column (Agilent, 30 m × 0.25 mm × 0.25 µm). Hydrogen was used as the carrier gas at a flow rate of 2 mL/min. The column was held at 170 °C for 2 min, ramped from 170 to 220 °C at 3 °C/min and held at 220 °C for 10 min. The content of monosaccharides was calculated using meso-erythritol (Sigma-Aldrich, St. Louis, Mo, USA) as an internal standard.

The content of uronic acids was determined by the sulfamate/3-phenylphenol colorimetric method [65] modified by Kim and Carpita [66].

### 4.4. Solid State ^13^C Nuclear Magnetic Resonance Spectroscopy

Cross-polarization magic angle spinning (CP MAS) NMR experiments were performed on a 400 MHz Bruker Avance III spectrometer, operating at 400.15 MHz and 100.63 MHz for ^1^H and ^13^C, respectively, equipped with a Broad Band (^15^N-^31^P) 4 mm MAS probe head. A sample of U-^13^C, ^15^N-labeled histidine hydrochloride was used to set the Hartmann–Hahn condition. The ^13^C CP MAS spectra were recorded with a proton 90° pulse length of 4 μs, 2 ms contact time, 6 s repetition time, and SPINAL64 decoupling (83 kHz amplitude).

Powdered WEP samples were packed into 4 mm ZrO_2_ rotor and spun at a spinning rate of 8 kHz. Due to the limited amount of material for study, long-term acquisition was necessary to achieve an acceptable signal-to-noise ratio in spectra, the 20 K transients were acquired. FIDs were accumulated using time domain size of 3.6 K data points. Adamantane (resonances at 38.48 and 29.46 ppm) was used as a secondary ^13^C chemical-shift reference from external tetramethylsilane (TMS). The NMR data were processed using Bruker Topspin software version 3.5.

### 4.5. Multi-Detection High-Performance Size-Exclusion Chromatography

The solutions of CWPs fractions (final retentates obtained after dialysis, Supporting Figure: Figure S1) were filtered through 0.45 μm syringe filters (Millipore Millex-HV, PVDF) and injected (100 µL) at 30 °C on a high-performance size-exclusion chromatography (HPSEC) system (Omnisec Resolve/Reveal, Malvern Panalytical), equipped with the following detectors: a refractive index (RI), laser low- and right-angle light scattering (LALS/RALS), differential capillary viscometer (DV), and photodiode array (UV-Vis). For the separation of root CW polymers, three serially connected Shodex OHpak SB-807, OHpak SB-806M, and OHpak SB-804 HQ columns (8 × 300 mm, Showa Denko K.K., Tokyo, Japan) with a guard column OHpak SB-807G (8 × 50 mm) were used. The samples were eluted at 1 mL/min with 50 mM NaNO_3_, containing 3 mM NaN_3_. The data were collected and analyzed using the OmniSEC software v11.41 (Malvern Panalytical, Malvern, UK) and d*n*/d*c* value of 0.146 mL/g. In each run, P-100 and P-800 pullulan standards (P-82 set, Showa Denko, Tokyo, Japan) were included to check the accuracy of analysis.

### 4.6. Statistics

All measurements were conducted at least in triplicate. Means were analyzed by one-way analysis of variance (ANOVA), using Statistica 13.3 software (Statsoft).

## 5. Conclusions

This study identifies HM AS domains within WEPs of triticale root apex as a structural marker of Al tolerance. Using multi-detection HPSEC with low- and right-angle light scattering detectors, we show that genotype-specific supramolecular assembly pattern of AS domain contributes to distinct responses under Al stress. The degree of symmetry observed in elongated lens-like AS pattern of root apical segments distinguishes the tolerant genotype from the sensitive one, which was ascribed to their contrasting supramolecular organization, revealed by imaging of a distribution of its macromolecular parameters by the multi-detection HPSEC system.

The AS domain from the apex of the tolerant genotype adopts branched flexible conformation with tight-fitting chain association, and thus may exclude the majority of Al cations under stress conditions. In this case, the AS domain virtually retains its initial shape, especially its longitudinal dimension, due to formation of separated subunits with higher molar mass, which can also block the spread of Al cations throughout the entire molecule. In contrast, AS domain of the sensitive genotype exhibits spherical flexible conformation with very loose structure related to weak associations between its chains, which can be easily infiltrated by Al cations. Therefore, Al stress induces formation of one completely integrated subunit with higher molar mass, but in this case, the AS domain undergoes severe destruction, including its longitudinal dimension.

The hairy root analogues display a micro gel structure with adsorption properties in both triticale genotypes. Nevertheless, Al stress positively affects the Al adsorption ability of the AS domain in the tolerant genotype, owing to its increased chain association, caused by a rise in polymer molar mass and feruloylation degree, while the adsorption ability of a counterpart of the sensitive one is decreased, as a result of the weakening of its chain interactions, which resulted from chain depolymerization induced by Al cations. Again, the marked differences in the initial supramolecular assembly of AS domains in both genotypes determine their opposed response to Al stress.

## Figures and Tables

**Table 1:** Content (%, dm) of neutral sugars and uronic acids (UA) and composition (mol%) of cell wall polysaccharides (CWPs) in the fractions sequentially extracted with water, CDTA, and Na_2_CO_3_ from apical and hairy root segments of the Al-sensitive (L438) and the Al-tolerant (L198) triticale genotypes and residue (RES) left after extraction under control and Al stress conditions.

Root Sample/Genotype	WEPs		CDTA-EPs		AEPs		RES	
	Control	Stress	Control	Stress	Control	Stress	Control	Stress
**Apical segment**								
**L438**								
Rhamnose	4.91 ± 0.22	4.43 ± 0.18	5.35 ± 0.23	3.86 ± 0.14	3.52 ± 0.08	3.14 ± 0.11	0.67 ± 0.04	0.64 ± 0.03
Fucose	1.81 ± 0.08	1.29 ± 0.06	2.21 ± 0.10	1.92 ± 0.08	1.13 ± 0.06	1.28 ± 0.04	0.28 ± 0.01	0.32 ± 0.01
Arabinose	26.08 ± 1.12	24.92 ± 1.20	25.9 ± 0.95	28.84 ± 1.31	26.00 ± 1.06	27.64 ± 1.33	22.64 ± 1.09	24.17 ± 1.19
Xylose	15.09 ± 0.68	13.87 ± 0.59	20.1 ± 0.91	19.8 ± 0.90	24.00 ± 0.99	19.14 ± 0.88	51.32 ± 1.95	49.05 ± 2.40
Mannose	4.35 ± 0.21	4.32 ± 0.15	3.23 ± 0.11	2.62 ± 0.10	3.54 ± 0.14	3.83 ± 0.18	0.49 ± 0.00	0.54 ± 0.01
Galactose	23.73 ± 0.98	27.06 ± 1.01	14.56 ± 0.71	15.37 ± 0.75	18.21 ± 0.54	20.52 ± 0.59	10.83 ± 0.51	13.36 ± 0.57
Glucose	16.17 ± 0.79	16.1 ± 0.59	23.78 ± 1.18	15.79 ± 0.45	17.76 ± 0.80	17.35 ± 0.86	9.8 ± 0.32	7.09 ± 0.25
Uronic acids	7.84 ± 0.35	8.00 ± 0.19	4.87 ± 0.23	11.8 ± 0.36	5.84 ± 0.25	7.11 ± 0.35	3.99 ± 0.18	4.86 ± 0.20
UA/Rha ^†^	1.60 ± 0.06	1.80 ± 0.07	0.91 ± 0.02	3.06 ± 0.14	1.65 ± 0.07	2.26 ± 0.06	5.96 ± 0.29	7.59 ± 0.29
CWP content ^‡^	22.49 ± 1.02	31.63 ± 1.56	10.85 ± 0.49	7.55 ± 0.37	15.41 ± 0.66	15.76 ± 0.63	39.26 ± 1.59	40.87 ± 1.91
**L198**								
Rhamnose	3.78 ± 0.18	3.29 ± 0.08	5.06 ± 0.24	3.25 ± 0.15	4.26 ± 0.19	2.89 ± 0.10	0.80 ± 0.05	0.60 ± 0.03
Fucose	1.74 ± 0.05	1.68 ± 0.07	2.56 ± 0.10	1.24 ± 0.05	1.24 ± 0.04	0.88 ± 0.04	0.34 ± 0.02	0.28 ± 0.00
Arabinose	28.4 ± 1.40	26.57 ± 1.31	23.76 ± 1.18	24.14 ± 1.19	24.72 ± 1.23	23.78 ± 1.11	22.21 ± 1.10	22.8 ± 1.03
Xylose	18.81 ± 0.84	16.38 ± 0.72	21.23 ± 1.01	19.24 ± 0.92	22.52 ± 1.05	29.36 ± 1.37	50.98 ± 2.15	51.75 ± 1.29
Mannose	2.91 ± 0.14	2.71 ± 0.13	3.33 ± 0.15	2.74 ± 0.13	3.49 ± 0.15	2.65 ± 0.08	0.54 ± 0.03	0.41 ± 0.01
Galactose	21.78 ± 0.92	24.65 ± 1.11	15.14 ± 0.69	13.87 ± 0.63	12.35 ± 0.51	14.56 ± 0.55	10.94 ± 0.45	11.35 ± 0.51
Glucose	16.68 ± 0.73	16.91 ± 0.54	21.68 ± 0.98	18.15 ± 0.85	22.29 ± 1.10	17.94 ± 0.89	8.91 ± 0.27	7.63 ± 0.15
Uronic acids	5.89 ± 0.19	7.82 ± 0.31	7.25 ± 0.27	17.35 ± 0.79	9.12 ± 0.42	7.95 ± 0.36	5.29 ± 0.21	5.19 ± 0.22
UA/Rha ^†^	1.56 ± 0.07	2.37 ± 0.10	1.43 ± 0.05	5.33 ± 0.24	2.14 ± 0.05	2.75 ± 0.09	6.61 ± 0.30	8.65 ± 0.41
CWP content ^‡^	32.91 ± 1.55	27.17 ± 1.06	13.94 ± 0.64	8.53 ± 0.42	15.18 ± 0.73	23.1 ± 1.02	32.21 ± 1.79	39.45 ± 1.53
**Hairy root segment**								
**L438**								
Rhamnose	8.49 ± 0.53	8.10 ± 0.61	7.09 ± 0.50	7.45 ± 0.43	5.82 ± 0.45	6.89 ± 0.38	0.82 ± 0.05	0.92 ± 0.05
Fucose	2.64 ± 0.18	2.61 ± 0.20	2.21 ± 0.19	2.35 ± 0.12	1.94 ± 0.12	2.33 ± 0.16	0.29 ± 0.02	0.35 ± 0.05
Arabinose	22.64 ± 1.54	19.71 ± 1.35	25.43 ± 1.08	22.65 ± 1.18	31.57 ± 2.03	25.86 ± 1.75	20.23 ± 1.22	20.88 ± 1.36
Xylose	11.18 ± 0.68	11.59 ± 0.85	15.60 ± 0.95	18.11 ± 0.73	22.4 ±1.42	19.27 ± 0.89	52.51 ± 3.05	51.98 ± 3.51
Mannose	7.23 ± 0.51	7.79 ± 0.52	4.68 ± 0.36	5.31 ± 0.33	3.08 ± 0.21	4.43 ± 0.32	0.65 ± 0.05	0.82 ± 0.07
Galactose	27.99 ± 1.59	29.36 ± 1.85	19.35 ± 1.29	22.4 ± 1.09	17.11 ± 1.01	24.28 ± 1.23	11.32 ± 0.78	11.65 ± 0.86
Glucose	12.03 ± 0.84	12.78 ± 0.74	12.46 ± 0.81	9.97 ± 0.85	8.87 ± 0.63	8.84 ± 0.56	9.72 ± 0.59	8.43 ± 0.49
Uronic acids	7.80 ± 0.56	8.06 ± 0.56	13.18 ± 0.95	11.77 ± 0.42	9.22 ± 0.49	8.10 ± 0.49	4.46 ± 0.36	4.97 ± 0.38
UA/Rha ^†^	0.91 ± 0.06	1.00 ± 0.09	1.86 ± 0.14	1.58 ± 0.11	1.58 ± 0.11	1.18 ± 0.09	5.44 ± 0.41	5.40 ± 0.35
CWP content ^‡^	34.62 ± 2.02	30.78 ± 2.10	6.07 ± 0.48	7.73 ± 0.56	15.4 ± 1.11	7.90 ± 0.59	36.74 ± 1.96	37.84 ± 2.14
**L198**								
Rhamnose	6.70 ± 0.41	7.74 ± 0.54	7.06 ± 0.43	7.23 ± 0.51	6.55 ± 0.46	4.87 ± 0.31	0.71 ± 0.05	0.62 ± 0.05
Fucose	3.43 ± 0.26	3.37 ± 0.20	3.12 ± 0.22	2.99 ± 0.12	2.49 ± 0.15	1.98 ± 0.16	0.30 ± 0.02	0.29 ± 0.02
Arabinose	25.51 ± 1.63	27.79 ± 1.88	26.84 ± 1.72	27.58 ±1.13	27.02 ± 1.88	26.55 ± 1.52	19.73 ± 1.14	19.55 ± 1.29
Xylose	19.07 ± 1.26	11.08 ± 0.65	21.84 ± 1.14	15.42 ± 0.87	22.51 ± 1.42	22.31 ± 1.46	55.26 ± 2.85	53.2 ± 2.55
Mannose	3.61 ± 0.25	3.56 ± 0.25	3.05 ± 0.21	3.37 ± 0.24	2.61 ± 0.18	2.83 ± 0.15	0.50 ± 0.04	0.39 ± 0.03
Galactose	24.61 ± 1.53	30.21 ± 2.05	17.6 ± 1.08	22.67 ± 1.33	20.7 ± 1.12	26.14 ± 0.91	11.75 ± 0.73	12.33 ± 0.77
Glucose	9.10 ± 0.64	8.19 ± 0.54	8.72 ± 0.51	8.79 ± 0.63	7.65 ± 0.45	8.44 ± 0.53	8.00 ± 0.47	9.20 ± 0.56
Uronic acids	7.96 ± 0.60	8.06 ± 0.49	11.77 ± 0.73	11.95 ± 0.66	10.47 ± 0.63	6.90 ± 0.49	3.75 ± 0.26	4.43 ± 0.35
UA/Rha ^†^	1.19 ± 0.08	1.04 ± 0.07	1.67 ± 0.12	1.65 ± 0.16	1.75 ± 0.08	1.42 ± 0.10	5.28 ± 0.39	7.14 ± 0.52
CWP content ^‡^	32.15 ± 2.18	26.44 ± 1.73	6.97 ± 0.43	8.48 ± 0.50	9.07 ± 0.64	11.45 ± 0.84	36.25 ± 2.06	38.37 ± 2.31

Data are represented by the mean ± SD of three replicates. ^†^ UA/Rha, a ratio of uronic acids to rhamnose. ^‡^ Sum of neutral sugars and uronic acids, expressed as % dm of each cell wall fraction isolated.

**Table 2:** Changes induced by Al stress in yield and macromolecular parameters of high and low molar mass (HM and LM) subunits of **WEP** fractions isolated from apical segments and hairy root samples of the Al-sensitive (L438) and the Al-tolerant (L198) triticale genotypes.

Sample/Genotype	Subunit	Yield ^†^ (%)	*M*_w_ (kDa)	*M*_w_/*M*_n_	[*η*] (mL g^–1^)	*R*_g_ (nm)	*R*_h_ (nm)	*R*_g_/*R*_h_	M-H a
**Apical segment**									
L438 control	HM-A	3.0	3330	1.03	1375	101	116	1.17	NC
	HM-B	16.5	2528	1.19	365	53	67	0.79	0.86
	LM-C	31.9	226	1.27	65	45	12	3.75	1.15
	LM-D	48.6	66	1.25	10	23	4	5.75	1.02
L438 stress	HM-A	2.5	4452	1.04	1657	105	126	0.83	NC
	HM-B	16.7	4333	1.20	360	50	72	0.69	0.83
	LM-C	37.1	367	1.29	53	51	14	3.64	1.11
	LM-D	43.7	117	1.23	15	53	13	4.08	1.00
L198 control	HM-A	2.6	3461	1.00	1431	134	100	1.35	NC
	HM-B	18.9	1573	1.49	346	59	43	1.37	0.54
	LM-C	39.4	164	1.24	61	55	11	4.72	1.24
	LM-D	38.1	44	1.17	12	27	6	4.50	0.98
L198 stress	HM-A	2.0	6086	1.03	1223	122	103	1.18	NC
	HM-B	14.1	4683	1.29	349	57	55	1.04	0.61
	LM-C	35.4	299	1.33	47	55	13	4.23	1.16
	LM-D	48.5	57	1.19	12	52	11	4.73	0.95
**Hairy root segment**									
L438 control	HM-A	5.9	7129	1.03	1531	103	111	0.93	NC
	HM-B	17.4	8058	1.12	521	55	89	0.62 (0.14) ^‡^	1.06
	LM-C	31.2	692	1.20	64	54	19	2.84	1.06
	LM-D	45.5	221	1.18	22	55	11	5.00	1.22
L438 stress	HM-A	5.0	5058	1.03	1849	117	109	1.07	NC
	HM-B	17.3	6145	1.12	539	56	80	0.70 (0.16) ^‡^	1.21
	LM-C	32.2	563	1.22	61	53	17	1.15	1.21
	LM-D	45.5	171	1.17	19	56	10	5.6	1.89
L198 control	HM-A	5.5	10,100	1.03	1561	85	141	0.60	NC
	HM-B	20.9	7963	1.26	399	53	80	0.66 (0.32) ^‡^	0.75
	LM-C	35.0	882	1.18	64	63	20	3.15	1.13
	LM-D	38.6	360	1.11	22	66	10	6.6	1.03
L198 stress	HM-A	4.5	13,800	1.05	1575	94	147	0.64	NC
	HM-B	19.7	11,400	1.16	395	53	91	0.58 (0.58) ^‡^	0.80
	LM-C	37.3	1250	1.18	54	61	21	2.90	1.94
	LM-D	38.5	506	1.19	21	64	11	5.82	1.91

^†^ Based on total amount of material recovered. ^‡^ Degree of feruloylation (in parenthesis) = (UV325 peak area/RI peak area). Values are means of at least four replicate analyses. Coefficients of variation < 7%. NC, not calculated. Peak limits for A and B subunits are 18–22 and 22–26 mL, respectively, and for C and D subunits, 26–30 and 30–34 mL. The concentration of HM subunits was 0.030–0.041 and 0.105–0.160 mg mL^–1^, for HM-A and HM-B, respectively.

## Data Availability

The original contributions presented in this study are included in the article/Supplementary Materials. Further inquiries can be directed to the corresponding author.

## Supplementary Materials

The following supporting information can be downloaded at https://www.mdpi.com/article/10.3390/ijms262311519/s1.
