# Supplementary material for: Supramolecular Assembly of Cell Wall Anisotropic Scatterers in Triticale Root Apex Reflects Aluminum Stress Response in Contrasting Genotypes
Source: Int J Mol Sci. 2025 Nov 27;26(23):11519. doi: 10.3390/ijms262311519 (PMC12692437; doi:10.3390/ijms262311519)
Supplement: Supplementary file 1 [file ijms-26-11519-s001.zip › ijms-3980569-supplementary.pdf]

Supplementary materials

Table S1: Changes induced by Al stress in yield and macromolecular parameters of subunits from CDTA-EP fractions;

Table S2: Changes induced by Al stress in yield and macromolecular param-eters of subunits from AEP fractions;

Figure S1: Superimposed HPSEC chromatograms of WEP, CDTA-EP and AEP fractions from apical segments as detected by UV254 nm and UV325 nm;

Figure S2: Superimposed HPSEC chromatograms of WEP, CDTA-EP and AEP fractions from hairy root segments as detected by UV254 nm and UV325 nm;

Figure S3: Superimposed multi-detector HPSEC chromatograms of CDTA-EP fractions;

Figure S4: Superimposed multi-detector HPSEC chromatograms of AEP fractions. See nomenclature in section 2.1;

Figure S5: Scheme of a sequential extraction of triticale root samples with water, CDTA and Na_2CO_3.

Table S1. Changes induced by Al stress in yield and macromolecular parameters of high and low molar mass (HM and LM) subunits of **CDTA-EP** fractions isolated from apical segments and hairy root samples of the Al-sensitive (L438) and the Al-tolerant (L198) triticale genotypes

Sample/ genotype	Subunit	Yield† (%)	M_w (kDa)	M_w/M_n	[η] (mL g^{-1})	R_g (nm)	R_h (nm)	R_g/R_h	M-H a
**Apical segment**									
L438 control	HM-A	0.24	5 028	1.39	578	NC	76	NC	NC
	HM-B	5.69	6 333	1.65	204	47	56	0.84	0.74
	LM-C	6.97	643	1.05	22	53	12	4.42	1.71
	LM-D	87.1	30	2.58	4	NC	2	NC	0.90
L438 stress	HM-A	0.41	5 344	1.28	708	98	81	1.21	NC
	HM-B	7.85	7 514	1.18	245	48	65	0.74	0.67
	LM-C	23.12	593	1.36	38	49	15	3.27	1.14
	LM-D	68.56	58	1.84	8	39	4	9.75	0.89
L198 control	HM-A	0.72	2 286	1.23	372	120	54	2.22	NC
	HM-B	5.26	4 438	1.31	230	50	53	0.98	0.47
	LM-C	12.68	407	1.32	45	53	14	3.78	1.09
	LM-D	81.34	20	2.11	7	NC	3	NC	0.88
L198 stress	HM-A	0.49	3 474	1.23	489	96	63	1.52	NC
	HM-B	5.55	5 743	1.37	195	46	54	0.87	0.44
	LM-C	17.91	339	1.34	37	13	12	1.08	1.45
	LM-D	76.05	30	2.07	7	NC	3	NC	0.92
**Hairy root segment**									
L438 control	HM-A	0.48	12 700	1.28	1 084	95	126	0.75	1.52
	HM-B	6.02	15 300	1.16	190	48	76	0.63	0.85
	LM-C	5.49	2 971	1.18	NC	63	NC	NC	NC
	LM-D	88.01	57	2.88	4	76	4	19.00	1.04
L438 stress	HM-A	0.70	14 900	1.05	942	70	129	0.54	1.75
	HM-B	5.84	13 800	1.16	210	48	76	0.63 (0.90)‡	0.86
	LM-C	9.77	1 908	1.24	NC	56	NC	NC	NC
	LM-D	83.7	80	2.07	5	56	4	14.00	1.14
L198 control	HM-A	0.50	15 200	1.11	1 115	82	136	0.60	0.50
	HM-B	4.58	11 200	1.40	227	51	72	0.71	0.79
	LM-C	6.48	1 655	1.14	NC	62	NC	NC	NC
	LM-D	88.44	45	2.44	3	56	3	18.67	1.21
L198 stress	HM-A	0.89	17 600	1.10	808	77	129	0.60	0.67
	HM-B	9.06	16 300	1.20	159	48	73	0.66 (1.29)‡	0.55
	LM-C	10.11	2 924	1.16	40	59	26	2.27	1.84
	LM-D	79.94	148	2.38	6	62	5	12.40	1.42

† Based on total amount of material recovered.
‡ Degree of feruloylation (in parenthesis) = (UV325 peak area/RI peak area).
Values are means of at least four replicate analyses. Coefficients of variation < 6%. NC, not calculated. Peak limits for A and B subunits are 18–22 and 22–26 mL, respectively, and for C and D subunits are 26–30 and 30–34 mL. The concentration of HM subunits was 0.003–0.004 and 0.015–0.018 mg mL^{-1}, for HM-A and HM-B, respectively.

Table S2 Changes induced by Al stress in yield and macromolecular parameters of high and low molar mass (HM and LM) subunits of **AEP** fractions isolated from apical segments and hairy root samples of the Al-sensitive (L438) and the Al-tolerant (L198) triticale genotypes

Sample/ genotype	Subunit	Yield† (%)	M_w (kDa)	M_w/M_n	[η] (mL g^{-1})	R_g (nm)	R_h (nm)	R_g/R_h	M-H a
**Apical segment**									
L438 control	HM-A	0.78	10 100	1.01	165	74	137	0.54	NC
	HM-B	12.89	48 100	1.07	175	51.3	109	0.47	0.48
	LM-C	7.30	26 800	1.02	64	56.2	56	1.00	1.35
	LM-D	79.03	1 436	2.79	3	58.1	6	9.68	NC
L438 stress	HM-A	3.42	10 500	1.00	199	66	132	0.50	NC
	HM-B	27.17	32 200	1.05	107	47	79	0.59	0.83
	LM-C	13.10	19 900	1.04	55	57	48	1.19	1.64
	LM-D	56.31	1 211	1.93	12	65	12	5.42	NC
L198 control	HM-A	0.32	6 312	3.80	222	74	59	1.25	NC
	HM-B	7.83	6 190	1.26	188	63	56	1.13	0.49
	LM-C	10.73	2 032	1.02	51	67	22	3.05	1.89
	LM-D	81.12	141	2.72	5	72	4	18.0	NC
L198 stress	HM-A	0.39	44 700	1.00	280	NC	NC	NC	NC
	HM-B	8.03	22 800	1.23	201	54	88	1.61	0.52
	LM-C	10.52	5 634	1.07	57	60	26	2.31	1.92
	LM-D	81.06	368	1.08	4	63	5	12.6	NC
**Hairy root segment**									
L438 control	HM-A	1.39	37 300	1.12	1 000	65	173	0.38	1.08
	HM-B	15.77	37 000	1.04	97	45	81	0.56 (1.34)‡	1.57
	LM-C	4.86	19 300	1.03	83	56	63	0.89	1.12
	LM-D	77.97	520	2.25	6	58	8	7.25	1.04
L438 stress	HM-A	0.96	38 200	1.12	1 083	70	179	0.39	1.70
	HM-B	16.83	34 100	1.05	79	45	74	0.61 (1.35)‡	1.48
	LM-C	6.23	15 900	1.07	72	56	56	1.00	1.05
	LM-D	75.98	567	2.16	5	60	7	8.57	1.00
L198 control	HM-A	0.67	47 100	1.12	726	69	169	0.41	1.86
	HM-B	13.04	33 000	1.06	81	47	77	0.61 (1.35)‡	1.07
	LM-C	3.71	14 500	1.05	77	57	56	1.02	0.78
	LM-D	82.57	382	2.18	4	61	6	10.17	1.03
L198 stress	HM-A	1.10	45 400	1.08	690	69	164	0.42	1.39
	HM-B	20.12	34 500	1.06	60	45	66	0.68 (1.35)‡	1.34
	LM-C	7.04	14 900	1.05	64	55	53	1.04	1.03
	LM-D	71.73	693	2.12	5	55	8	6.88	1.04

† Based on total amount of material recovered.
‡ Degree of feruloylation (in parenthesis) = (UV325 peak area/RI peak area).
Values are means of at least four replicate analyses. Coefficients of variation < 7%. NC, not calculated. Peak limits for A and B subunits are 18–22 and 22–26 mL, respectively, and for C and D subunits are 26–30 and 30–34 mL. The concentration of HM subunits was 0.003–0.006 and 0.040–0.070 mg mL^{-1}, for HM-A and HM-B, respectively.

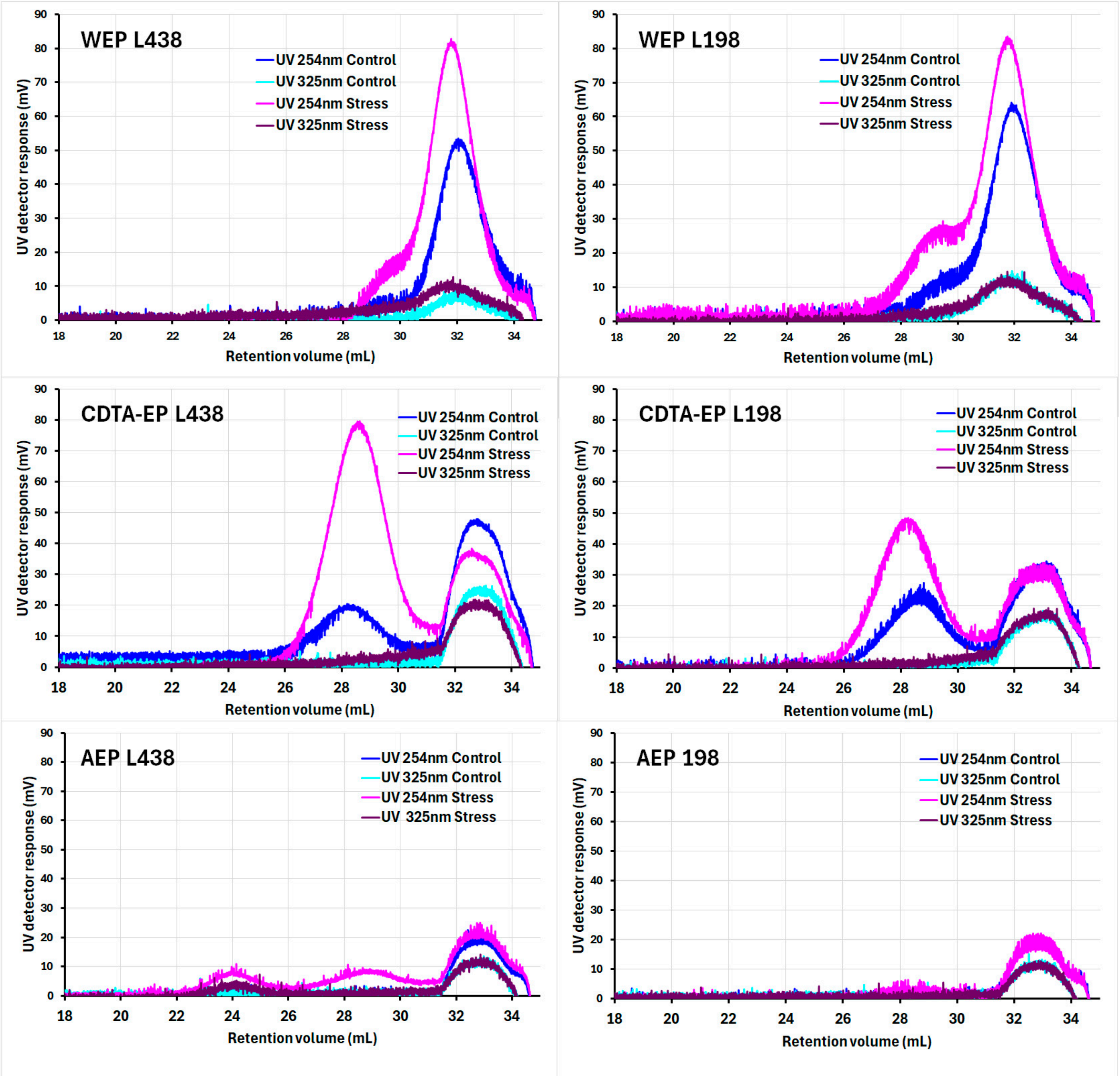

Figure S1. Superimposed HPSEC chromatograms of WEP, CDTA-EP and AEP fractions isolated from apical segments of the Al-sensitive (L438) and the Al-tolerant (L198) triticale genotypes as detected by UV254 nm and UV325 nm. For interpretation of the references to colour in this figure legend, the reader is referred to the web version of this article.

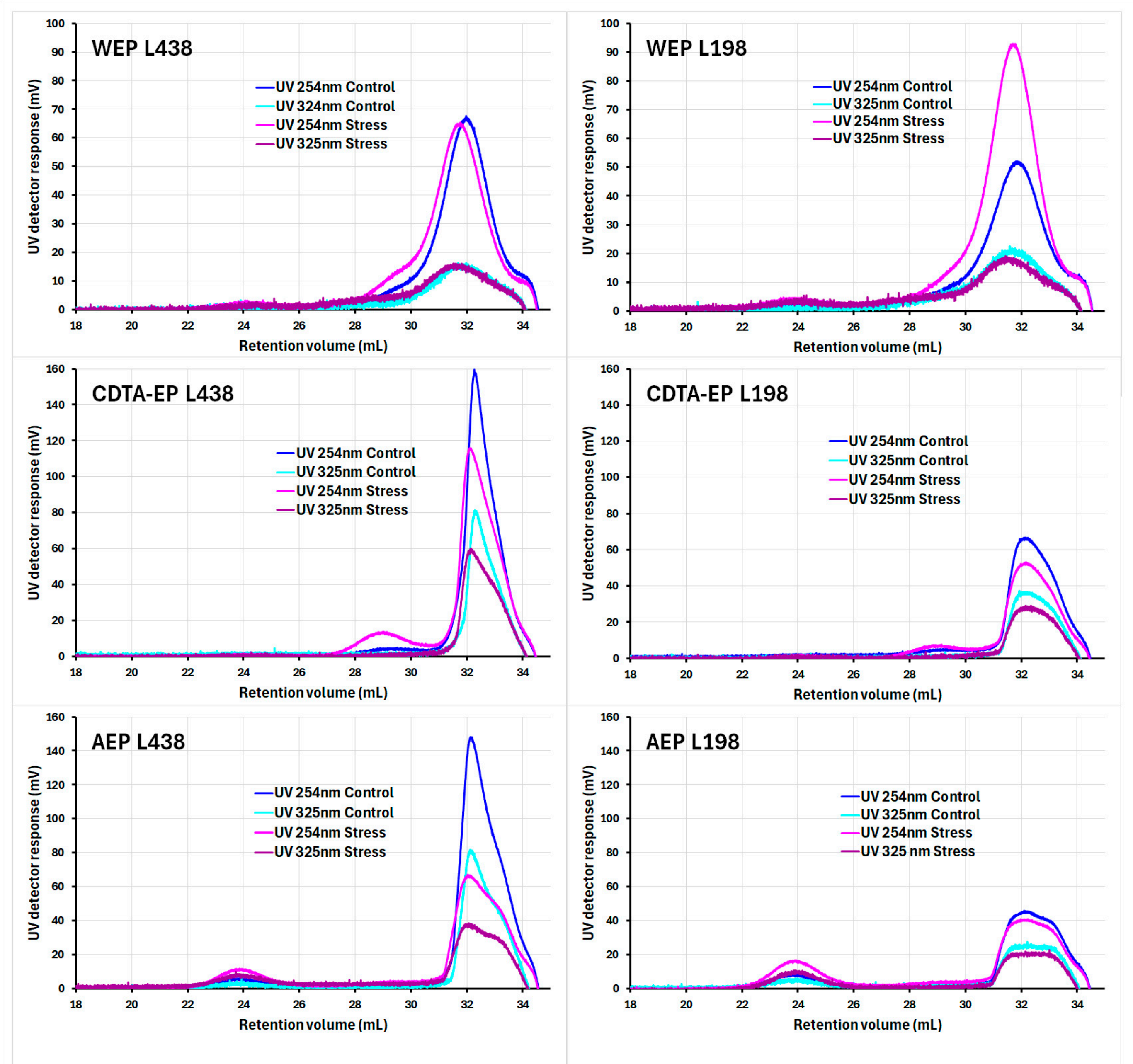

Figure S2. Superimposed HPSEC chromatograms of WEP, CDTA-EP and AEP fractions isolated from hairy root segments of the Al-sensitive (L438) and the Al-tolerant (L198) triticale genotypes as detected by UV254 nm and UV325 nm. For interpretation of the references to colour in this figure legend, the reader is referred to the web version of this article.

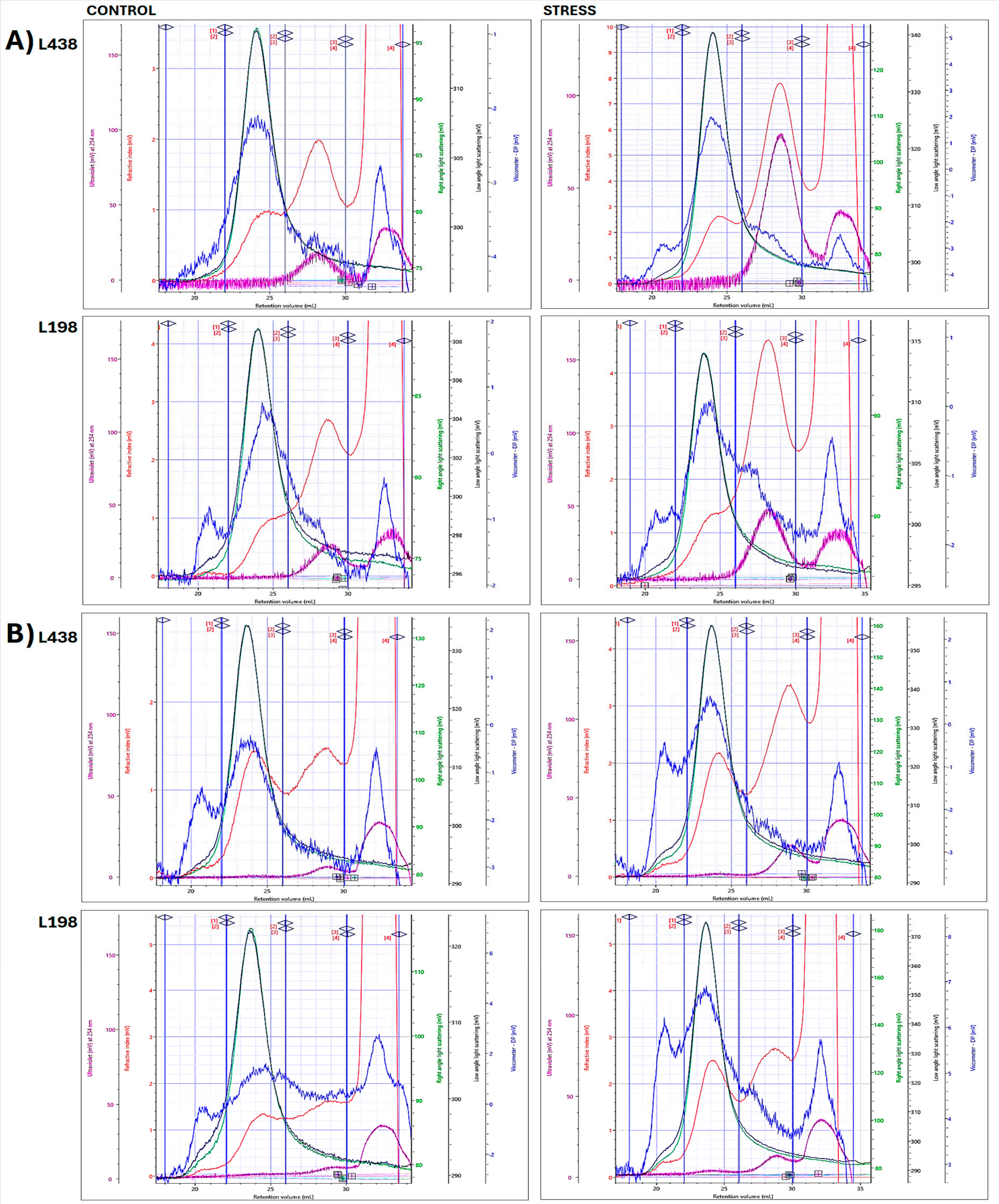

Figure S3. Superimposed multi-detector HPSEC chromatograms of **CDTA-EP** fractions isolated from **(A)** apical segments and **(B)** hairy root samples of the Al-sensitive (L438) and the Al-tolerant (L198) triticale genotypes as detected by five detectors: RI (**red line**), DV (**blue line**), RALS (**green line**), LALS (**black line**) and UV (**purple line**). The signals of all detectors have been scaled to improve visualization. For interpretation of the references to colour in this figure legend, the reader is referred to the web version of this article.

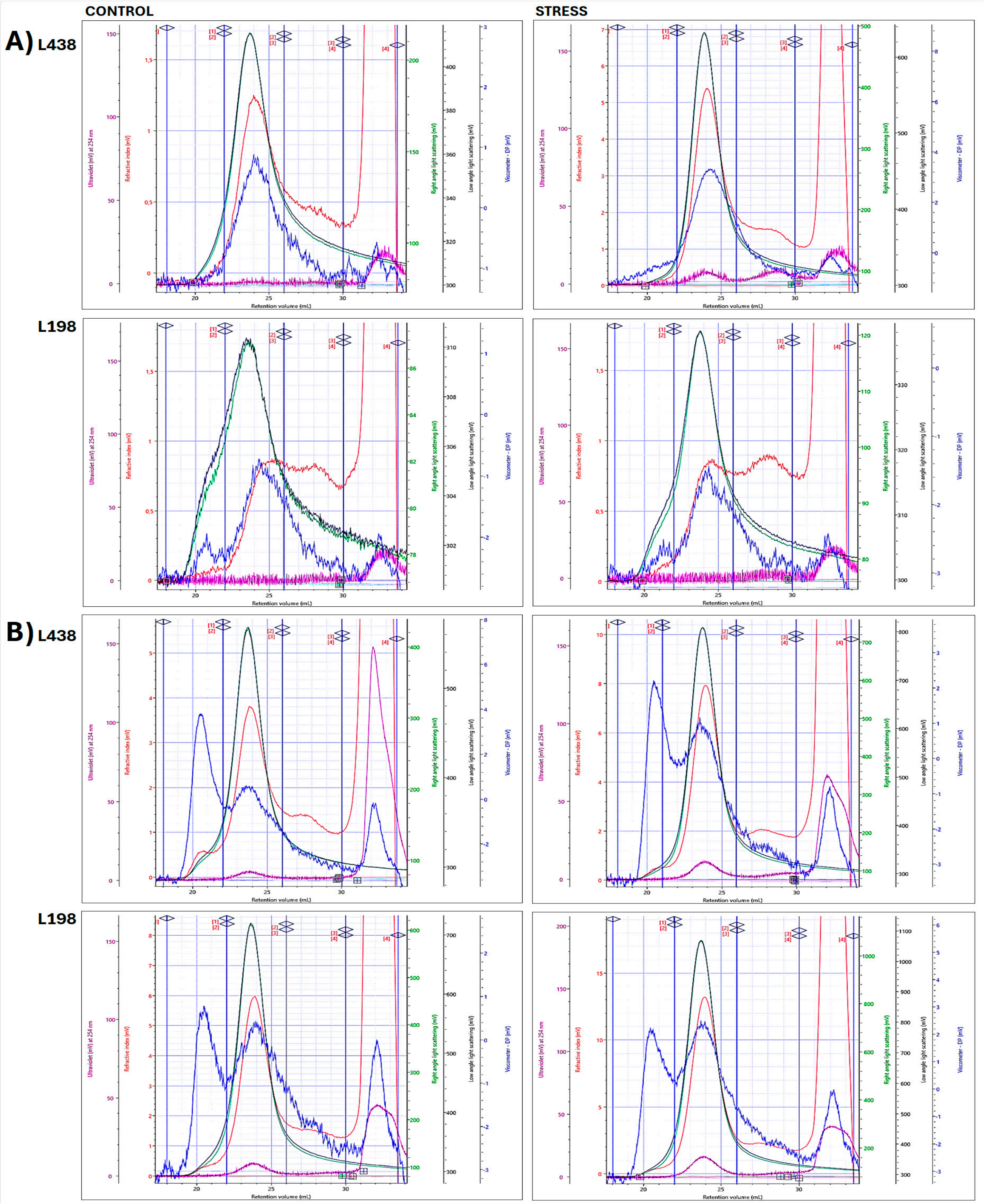

Figure S4. Superimposed multi-detector HPSEC chromatograms of **AEP** fractions isolated from **(A)** apical segments and **(B)** hairy root samples of the Al-sensitive (L438) and the Al-tolerant (L198) triticale genotypes as detected by five detectors: RI (**red line**), DV (**blue line**), RALS (**green line**), LALS (**black line**) and UV (**purple line**). The signals of all detectors have been scaled to improve visualization. For interpretation of the references to colour in this figure legend, the reader is referred to the web version of this article.

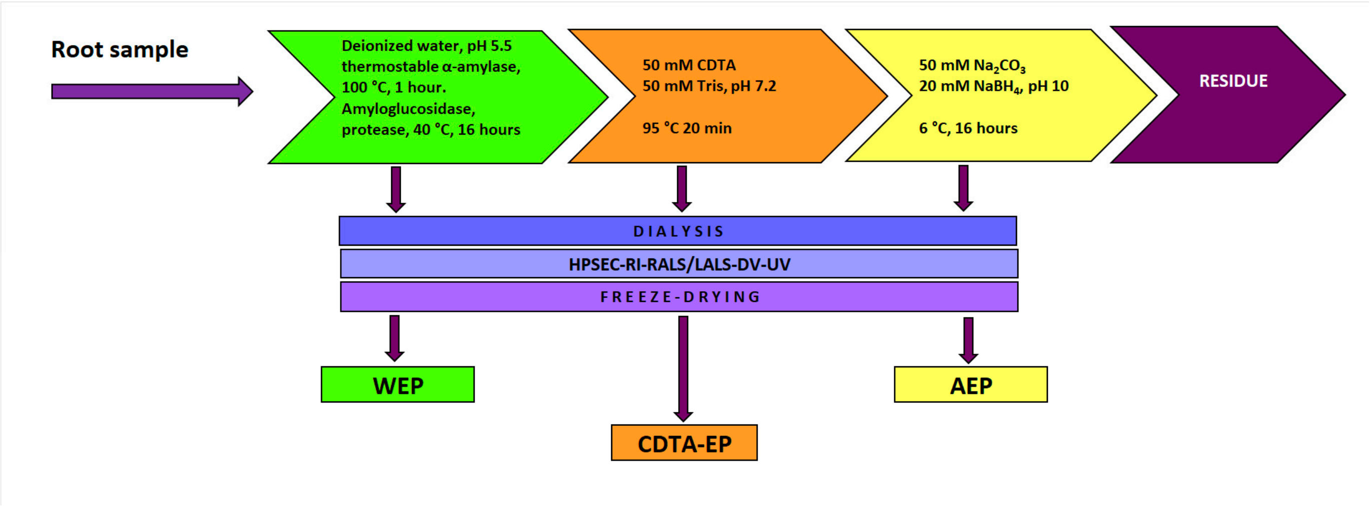

Figure S5. Scheme of a sequential extraction of triticale root samples with water, CDTA and Na_2CO_3 solutions and isolation of water-, CDTA- and alkali-extractable cell wall polysaccharide fractions (WEP, CDTA-EP and AEP, respectively).
